# Time to birth for spontaneous and ART births among infertile groups

**DOI:** 10.1186/s12905-025-04016-3

**Published:** 2025-10-28

**Authors:** Marie-Caroline Compans, Eva Beaujouan, Vegard Skirbekk

**Affiliations:** 1 University of Vienna (Wittgenstein Centre for Demography and Global Human Capital – IIASA, OeAW, University of Vienna), Vienna, Austria; 2 Institut national d’études démographiques (Ined), Aubervilliers, France; 3 Center for Fertility and Health, Norwegian Institute of Public Health, Oslo University, Oslo, Norway; 4 Columbia University, New York, NY USA

**Keywords:** Assisted reproduction, Time to birth, Infertility, Norway, Determinants of birth timing

## Abstract

**Background:**

Childbearing postponement in high-income countries has likely led to an increase in the incidence of age-related infertility. While assisted reproductive technologies (ART) can help some individuals to become parents, this often takes time. However, we know little about how long it takes individuals with infertility problems to achieve a live birth, whether they use ART or not, and how this has changed over time.

**Method:**

We use retrospective information from the HUNT study, which provides rare information on the age at first conception attempt among people who have ever experienced infertility issues. These data are linked to the Norwegian Medical Birth Registry (NMBR), which allows tracking the timing of live births and whether they result from ART. We control for several maternal and paternal characteristics and distinguish between the late 1980–1990s (1987–99) and the 2000–2010s (2000–19). Cox models were fitted to samples of 863 mothers and 590 fathers.

**Results:**

The average time from the start of conception attempt to spontaneous births is three years, and to ART births is five years. ART births occurred faster when pregnancy attempts were initiated in 2000–19 rather than 1987–99 (coeff = 0.536, [0.198; 0.873] for women, coeff = 0.487, [0.072; 0.902] for men), and when men and women tried to conceive for the first time at age 35–39 (coeff = 1.215, [0.260; 2.171] for women, coeff = 1.005, [0.275; 1.735] for men). This was not the case for spontaneous births.

**Conclusions:**

While assisted reproductive technologies enable individuals facing infertility issues to achieve a live birth, they require more time to succeed. However, since the 2000s, time to ART births has decreased, with the shortest observed among those initiating conception attempts in their late thirties. Despite the fact that age remains a significant negative factor associated with the declining occurrence of births, these findings may reflect shifts in the profile of fertility clinic patients, faster care-seeking processes, and improvements in ART efficiency.

## Introduction

**Fig. 1:**
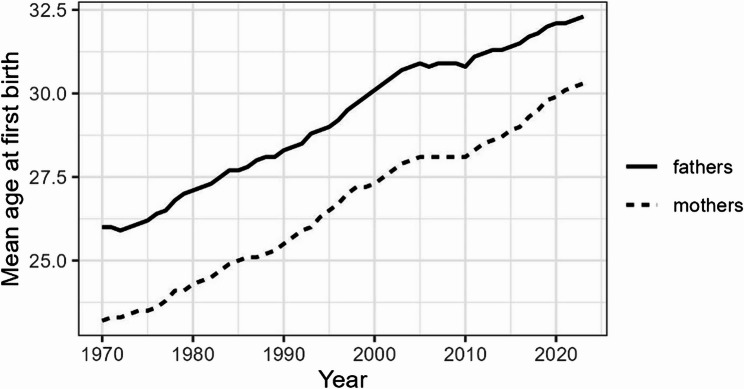
Average age at first birth, men and women, Norway, 1970–2023. Authors’ calculations. Source: Statistics Norway (2023)

In recent decades, childbearing has been increasingly delayed in high-income countries, driven by changes in marriage and partnership, social values, and the labour market [1]. In Norway, since 2020, the mean age at first birth has been above 30 years for women and 32 years for men (Fig. 1). The country has many characteristics that are also specific to late childbearing, including high educational attainment [2], late age at marriage [3], high female employment rates [4], rising housing prices [5], and rapid secularisation in recent decades [6].

As reproductive capabilities tend to decrease when conception attempts are delayed [7], this general trend towards later family formation may have led to an increase in the prevalence of infertility, medically defined as unsuccessful conception attempts for more than 12 months. Because the treatments became more widely accepted and as the average age of women seeking to have children increased, the demand for infertility treatments may have thus increased [8–10].

**Fig. 2:**
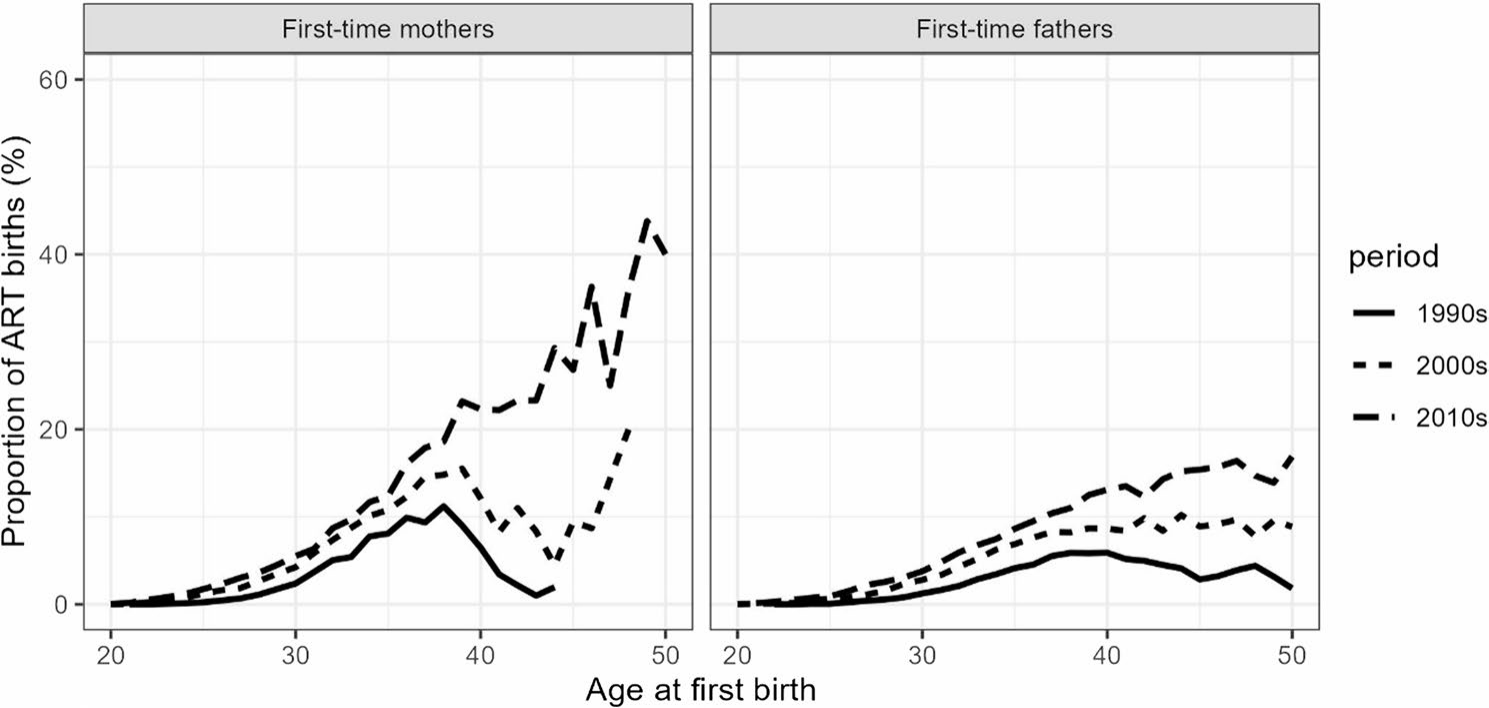
Share of ART births among first births by maternal and paternal age and period. Authors’ calculations. Source: Norwegian Medical Birth Register (1990–2019)

ART in Norway is publicly covered for up to three cycles, and until 2020, there was no age limit. From 2020, new regulations imposed an upper age limit of 46 years for ART treatments for women while allowing egg donation and ART access for single women [11]. Since the birth of the first Norwegian ‘test-tube’ baby in 1984, assisted reproductive technologies (ART) have improved, with a significant impact on fertility. In 2014, 3.7% of births in Norway resulted from infertility treatments, a rise particularly visible at advanced maternal ages [8]. Until the 2000 s, ART contribution to first births was the highest before age 40; from the 2010s, it began to contribute more at age 40 and over (Fig. 2). For men, ART births increased to a lesser extent, but the rise was also substantial at late reproductive ages.

Although ART has the potential to help individuals who have difficulties conceiving, achieving a live birth through ART is not systematic and takes time [12]. In the general population, women who receive ART treatments generally take longer to have a child, primarily due to underlying subfertility issues [7, 12, 13]. Yet, focusing on people facing reproductive difficulties and who eventually have a child, little is known about the time to birth when succeeding or not via ART.

In addition, one might wonder whether ART births occur faster now than in the past. ART patients have tended to be older over time, which is likely to be associated with longer time to conception [8]. At the same time, more women may be using ART for age-related reasons [9], possibly suffering less severe infertility impairments and experiencing shorter times to birth. Technological improvements, notably pioneered by the Nordic countries, may also have reduced the time in treatment [14].

This paper leverages retrospective information from the HUNT study on age when trying to conceive for the first time among Norwegian women who have ever experienced infertility issues, information that is rarely available [15, 16]. We analyse how time to birth has changed over time for ART and spontaneous births, and the maternal and paternal factors associated with the time since trying to have a child for both conception modes.

## Material & method

### The HUNT study

The Trøndelag Health Study (HUNT) is one of the largest health studies ever conducted, which consists of four waves: 1984–86, 1995–99, 2006–08 and 2017–19. This population-based study collects health data and biological samples from the inhabitants of Nord-Trøndelag County, Norway. It involves a large proportion of the total population in the region, with tens of thousands of participants in each wave [17, 18]. The survey provides valuable data for numerous medical and public health research projects by collecting information on various aspects of health, including physical measurements, blood samples, information on lifestyle, general health and specific health conditions, prescriptions and medication use. It is one of the rare population-based studies that contains information on age at the time of trying to conceive for the first time [15, 16], allowing an analysis of changes over different periods. In addition, common unique identifiers between the HUNT study and population registries allow linkage to study changes in health patterns and risk factors.

### Main outcome of interest

This analysis examines maternal and paternal factors associated with the time from first conception attempt to first live birth among ever-infertile individuals. We use waves 2 (1995–99), 3 (2006–08) and 4 (2017–19) of the HUNT study, in which women aged 20–79 who have ever experienced infertility issues (i.e., those who answered yes to ‘Have you ever tried for more than one year to become pregnant?’) were asked about their age when they first tried to become pregnant (‘How old were you the first time you tried to become pregnant?’). Note that the first pregnancy attempt may not be the one for which women experienced difficulties conceiving. We exclude wave 1, where these questions were not asked. Responses were recorded as rounded ages. Using an individual identifier provided both in HUNT and the Norwegian Medical Birth Registry (NMBR), we retrieve from NMBR administrative information on live births from 1967 to 2020 for all HUNT survey participants. This linkage with NMBR also provides information on the timing of births and whether they resulted from ART treatment (in vitro fertilization or intracytoplasmic sperm injection). ART use is only known if it resulted in a live birth, but we do not know if people used infertility treatments unsuccessfully or if they conceived spontaneously despite using ART. For this reason, we focus on the outcome (ART or spontaneous births) irrespective of the pathway to conception, and we discuss this limitation at the end of the paper.

### Study sample for women

**Fig. 3:**
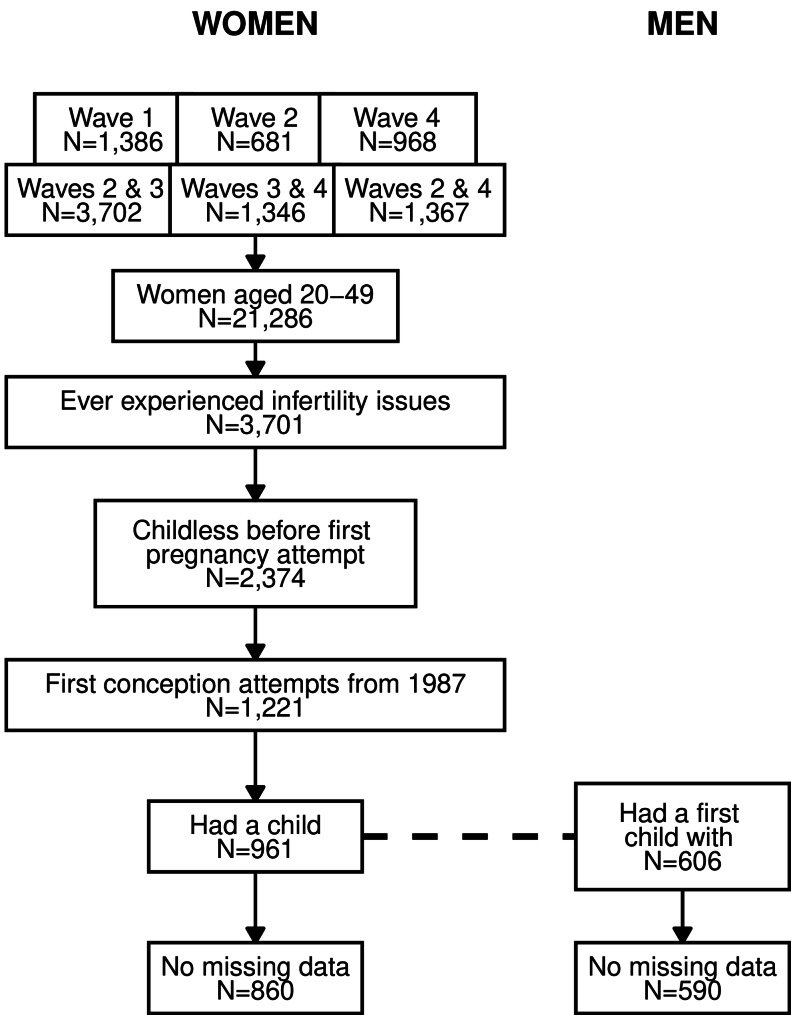
Selection of study samples of women and men in the HUNT study

In the studied HUNT waves (2, 3 and 4), 4,645 women were surveyed only once and 26,829 more than once (Fig. 3). To minimize recall biases [19], we selected women aged 20 to 49 at the time of the survey (*n* = 21,286).

We only focused on those who had ever experienced infertility issues (17%, *n* = 3,701). Overall, respondents who had been interviewed twice were consistent with their answers about pregnancy attempts across waves. In case of inconsistencies, we retained the youngest age at which women reported trying to conceive for the first time. To correct for age heaping at multiples of five, the distribution of age at the first pregnancy attempt was smoothed using a Gaussian kernel density estimator with a fixed bandwidth of two years [20].

Some women likely reported the age at which they experienced infertility rather than the age at which they first tried to conceive. Others may have had an unplanned first pregnancy. For these reasons (and possibly other unknown ones), we also excluded 1,427 women who already had a child before the reported age at first pregnancy attempt.

We do not include 1,053 mothers who tried to conceive before 1987, when ART activity was still nascent. In this sample, the proportion of women who became mothers decreased with age at initiating conception attempts (Table 1).

**Table 1:** Proportions of ever-infertile women who had a live birth by age at initiating conception attempts

	%	Standard error	CI (95%)
20–24	89.4	1.4	[86.7;92.1]
25–29	88.1	1.3	[85.6;90.6]
30–34	83.1	2.0	[79.2;87.0]
35–40	82.8	3.1	[76.7;88.9]
40+	78.9	7.4	[64.4;93.4]

Source: HUNT 2, 3, 4

Sample: Women aged 20 to 49 at the time of the survey, who have ever experienced infertility issues, childless at the age they reported at initiating conception attempts

Finally, as the analysis focuses on women who eventually became mothers, the sample excludes 260 childless women (13%).

### Study sample for men

For each birth, the NMBR provides the characteristics and unique identifier of both parents. Linkage between the HUNT study and the birth registry allows for the identification of births when both the mother and the father were survey participants. Therefore, we can focus on surveyed men who had their first child with a surveyed mother who had ever experienced infertility issues (*n* = 606 men). The age at which they first attempted to conceive is derived from the mother’s reported age at trying to become pregnant and the age difference between the parents. Although we do not have first-hand information on men’s experience of infertility and age at conception attempts, we assume that men first tried to become fathers and experienced infertility issues with the mother of their first child.

### Analytical strategy

We employ Cox models to estimate the duration between the year respondents initiated their pregnancy attempts and the year of first live birth. Ever-infertile individuals who used ART successfully may have different characteristics than those who conceive spontaneously (for instance, they may be more educated and older). For this reason, models are run separately for ART and spontaneous conceptions to examine how factors operate differently in relation to time to birth. This is estimated by the age at which they started trying to conceive (as categories). Models also include two periods for the first pregnancy attempt: 1987–99 and 2000–19. Based on data from HUNT, we control for health indicators (having ever smoked, the body mass index and chronic disease status in the year prior to the survey). From population registry linkages, we include controls for social disparities in childbearing schedules (educational attainment), intergenerational transmission of fertility behaviour (age of the respondent’s mother at her first birth, number of siblings), a binary indicator of family-proneness (marital status at the start of pregnancy attempts) and the age difference with the other parent. Excluding observations with missing data, models were run on 863 mothers and 590 fathers. We use the *survival* package in R. Variables for which the proportionality assumption was not met are interacted with time.

Table 2 displays the distribution of covariates in the study samples of mothers and fathers. We report the coefficients of the model. For covariates with constant hazards, a positive value indicates greater hazards, hence shorter time to birth. Coefficients for covariates with time-varying associations reflect whether their association with time to birth changes with time since trying to conceive. Positive values indicate that associations increase proportionally to log(t). We comment on results that are significant at the 5% level.

**Table 2:** Distribution of health and family background characteristics by sex

	Mothers	Fathers
First pregnancy attempts in 1987–99 (%)	70.5	67.5
Mean age at first pregnancy attempt (years)	25.3	28.6
ART births (%)	19.6	21.4
Tertiary educated (%)	51.1	32.4
Married at first pregnancy attempt (%)	32.3	34.9
Ever smoked (%)	49.8	45.8
Chronic disease status (%)	43.7	40.7
Mean BMI	27.9	28.6
Mean age difference with the partner (years)	3.1	4.1
Mean age of ego’s mother at her first child (years)	22.8	23.1
Mean number of siblings	2.1	2.3

Source: HUNT 2, 3, 4. Sample: Mothers who have ever experienced infertility issues, fathers who had their first child with a woman who has ever experienced infertility issues

## Results

### Descriptive results

In line with the general trend shown by the birth registry (Fig. 1), across the observed periods, an increasing proportion of parents who had ever experienced infertility issues had their first child through ART. It increased from 8 to 16% for mothers and from 9 to 21% for fathers between 1987–99 and 2000–19 (Table 3). Among mothers who reported infertility issues, those who eventually conceived spontaneously initiated their first pregnancy attempt at a slightly younger age on average (25.1 years) than those who conceived with medical help (25.9 years). For men who became fathers with a woman who ever had infertility issues, the first conception attempt with ART was a bit more delayed than with spontaneous conception (29.9 years vs. 28.3 years). Finally, first spontaneous live births occurred faster than ART births: three years vs. five years after the first conception attempt, on average.

**Table 3:** Age at trying to conceive for the first time, time to birth by type of conception and birth type by period at initiating conception attempts

	Mothers			Fathers		
	ART births	Spontaneous births	t-test/Chi2 test	ART births	Spontaneous births	t-test/Chi2 test
Mean age at trying to conceive for the first time (years)	25.9	25.1	*	29.9	28.3	***
Mean time to birth since trying to conceive for the first time (years)	5.1	2.9	***	5.0	2.9	***
Type of births by period when trying to conceive for the first time (%)						
1987–99	7.9	92.1	***	8.8	91.2	***
2000–19	15.9	84.1		21.4	78.6	

Source: HUNT 2, 3, 4. Sample: Mothers who have ever experienced infertility issues, fathers who had their first child with a woman who has ever experienced infertility issues

### Maternal and paternal factors of time to birth

Table 4 reports adjusted coefficients for female and male factors associated with time to birth since first trying to conceive among individuals who have ever experienced infertility issues. Holding other factors constant, for spontaneous conceptions, age at first attempt is not significantly associated with time to birth for mothers, while men in their thirties experience longer time to birth compared to men in their mid-twenties. Conversely, ART births occurred significantly faster among those who first tried to conceive at age 35–39 than in the other age groups for women (coeff = 1.215, [0.260; 2.171]) and men (coeff = 1.005, [0.275; 1.735]). Across periods, time to birth did not significantly change for spontaneous conception, but decreased for ART births (coeff = 0.536, [0.198; 0.873] for women, coeff = 0.487, [0.072; 0.902] for men).

**Table 4:** Estimated associations between time to birth since the initiation of conception attempts and maternal and paternal factors (Cox models)

	Mothers						Fathers					
	ART births			Spontaneous births			ART births			Spontaneous births		
	Coeff.	CI (95%)	*p*-value	Coeff.	CI (95%)	*p*-value	Coeff.	CI (95%)	*p*-value	Coeff.	CI (95%)	*p*-value
Age at trying to conceive (ref=’25–29’)												
< 20	−1.117	[−2.249;0.015]	0.053	−0.223	[−0.802;0.357]	0.451	−2.736	[−4.949;−0.524]	0.015	0.472	[−1.509;2.453]	0.641
20–24	−0.739	[−1.119;−0.358]	< 0.001	0.088	[−0.125;0.3]	0.420	−0.623	[−1.265;0.019]	0.057	−0.276	[−0.622;0.071]	0.119
30–34	0.101	[−0.434;0.635]	0.712	−0.033	[−0.33;0.264]	0.826	0.071	[−0.414;0.556]	0.774	−0.383	[−0.673;−0.094]	0.009
35–39	1.215	[0.260;2.171]	0.013	0.085	[−0.472;0.642]	0.764	1.005	[0.275;1.735]	0.007	−0.612	[−1.116;−0.108]	0.017
40+				−0.444	[−1.595;0.707]	0.449	0.420	[−0.569;1.409]	0.406	−0.248	[−1.043;0.548]	0.542
Trying to conceive in 2000–19 (ref=’1987–99’)	0.536	[0.198;0.873]	0.002	0.101	[−0.083;0.285]	0.280	0.487	[0.072;0.902]	0.021	0.171	[−0.066;0.408]	0.157
Tertiary educated	*0.041*	*[−0.090;0.173]*	*0.539*	0.029	[−0.137;0.196]	0.730	0.053	[−0.393;0.499]	0.816	0.266	[0.051;0.482]	0.015
BMI (ref=’18.5–24.9’)												
< 18.5	-	-	-	-	-	-	-	-	-	−0.281	[−2.285;1.723]	0.784
25–29.9	0.167	[−0.236;0.569]	0.417	−0.233	[−0.421;−0.045]	0.015	0.211	[−0.348;0.771]	0.459	0.179	[−0.086;0.443]	0.186
30+	−0.113	[−0.497;0.271]	0.564	−0.241	[−0.438;−0.045]	0.016	−0.042	[−0.684;0.600]	0.897	0.015	[−0.278;0.307]	0.921
Ever smoked	−0.104	[−0.448;0.241]	0.555	−0.197	[−0.356;−0.038]	0.015	0.022	[−0.363;0.407]	0.911	−0.102	[−0.292;0.088]	0.292
Chronic disease in the past year	−0.008	[−0.352;0.336]	0.963	0.026	[−0.132;0.183]	0.750	−0.138	[−0.515;0.239]	0.474	−0.100	[−0.298;0.098]	0.324
Age of ego’s mother at first birth	0.000	[−0.045;0.045]	1.000	*−0.001*	*[−0.003;0.001]*	*0.498*	−0.029	[−0.079;0.021]	0.260	−0.006	[−0.031;0.020]	0.674
Number of siblings	−0.104	[−0.253;0.044]	0.169	−0.013	[−0.081;0.056]	0.714	*0.010*	*[0.002;0.018]*	*0.012*	−0.056	[−0.134;0.022]	0.158
Married when starting to try to conceive	0.192	[−0.184;0.568]	0.317	0.019	[−0.251;0.288]	0.893	0.382	[−0.090;0.855]	0.113	−0.544	[−0.845;−0.244]	0.203
Age difference with the mother (ref=’Same age’)												
Woman is older (1+)	−0.577	[−1.257;0.103]	0.096	−0.430	[−0.727;−0.133]	0.005	−0.144	[−1.170;0.883]	0.784	−0.255	[−0.648;0.137]	0.203
Man is older (1–5 years)	−0.023	[−0.407;0.361]	0.906	−0.076	[−0.269;0.118]	0.443	−0.365	[−0.861;0.130]	0.148	−0.105	[−0.352;0.142]	0.403
Man is way older (6+)	−0.011	[−0.453;0.431]	0.961	−0.213	[−0.433;0.007]	0.058	−0.737	[−1.352;−0.122]	0.019	−0.169	[−0.478;0.141]	0.285
Married*Age at trying to conceive												
< 20				−0.218	[−0.921;0.485]	0.543				−1.284	[−3.744;1.177]	0.307
20–24				−0.371	[−0.738;−0.004]	0.048				−0.046	[−0.556;0.464]	0.860
30–34				−0.007	[−0.616;0.601]	0.982				0.530	[0.042;1.017]	0.033
35–39										0.928	[−0.054;1.909]	0.064
40+				−0.731	[−3.042;1.579]	0.535				−0.588	[−1.881;0.704]	0.372
Log Likelihood	55.7			47.8			41.6			50.5		
Mean time to birth (years)	5.15			2.94			5.03			2.88		
*N*	169			694			126			464		

- indicates too few observations for estimation. Numbers in italics refer to covariates that interact with time

Source: HUNT 2, 3, 4

Sample: Mothers who have ever experienced infertility issues, fathers who had their first child with a woman who has ever experienced infertility issues

Among other factors significantly associated with time to birth, women higher BMI and having ever smoked increased the time to live birth for spontaneous conception. These health factors were not significantly associated with the timing of ART births. Women who conceived spontaneously and were older than the father, and similarly, ART fathers who were more than five years older than the mothers, took significantly longer to have their first child. While the association between education and time to birth is not significant for women, tertiary-educated men spontaneously had a first child faster compared to their educational counterparts (coeff = 0.266, [0.051; 0.482]). Being married when trying to conceive (vs. not being married) is significantly associated with slightly longer time to spontaneous birth when women started trying at age 20–24 rather than at age 25–29 (0.019*0.088*−0.371=−0.001) and shorter time when men started trying to conceive at age 30–34 (0.530*−0.544*−0.383 = 0.110).

## Discussion

Our study shows that, when controlled for age at first birth, the time taken to conceive through ART has decreased between the 1990s and the 2000–2010s, whereas the time taken for spontaneous births has remained stable. This may indicate more efficient infertility treatments. In addition, even when focusing on individuals who have experienced infertility, ART births typically require a longer time span than spontaneous births. In view of the selection effects at play (e.g., conceiving before considering ART), this may be attributed to the particularly low chances of spontaneous conception of women who use ART. Hence the fact that ART births take longer than spontaneous births observed in the general population [7, 12, 13] is also evident in the subfertile population. Future studies could consider the mechanisms and selection effects involved in the decision to seek ART, as well as the influence of social or psychological factors such as stress or personal choice.

While age is negatively associated with achieving a live birth, both for spontaneous and ART births [7, 12], ART births occur earlier for men and women who start trying to conceive in their late thirties. Younger patients may have specific medical fertility issues that make it more difficult to conceive, whereas for patients over 40, slower conception could be due to biological reasons. Conversely, patients in their mid-thirties may seek ART more quickly once diagnosed with infertility issues, resulting in a swifter procedure. For men and women who have experienced infertility and have conceived spontaneously, age at the time of initiating conception attempts is not an important determinant. Instead, health factors, such as high BMI and smoking, are associated with a longer time to birth.

Our study has limitations. Despite our efforts to rule out response bias, the reliability of retrospective collection of age at initiating pregnancy attempt remains questionable. Such data do not provide information on the regularity of sexual activity and thus on risk exposure to pregnancy [15, 16]. In addition, trying to conceive is not linear, as some life course events may contribute to pauses in pregnancy attempts [13]. We also use an indirect measure of age when trying to conceive for men, derived from women’s responses and only when they become fathers with a woman of the studied sample. Hence, our study sample of men may be selected, which highlights the need for more comprehensive collection of data on men’s fertility. Another limitation is that we lack information on the timing of infertility and treatment use after trying to conceive for more than a year, and whether some couples were using ART but had a child spontaneously. As we only observe successful infertility treatments, our focus was on the time taken to give birth among people who had a child, according to the method of conception. We do not reflect the path to conception. We also did not have access to information on other types of infertility treatment, such as ovulation induction or intrauterine insemination. This could refine the analysis of the contribution of medically assisted reproduction to time to birth. Finally, the question of the generalizability of our findings to other contexts is left open by the lack of comparable data with information on age at conception attempts.

## Conclusion

Although assisted reproductive technologies enable infertile individuals to have a live birth, they take longer time to succeed. However, since the 2000s, time to ART birth has been decreasing, with the shortest span observed among those who initiate conception attempts in their late thirties. These trends are embedded in a context where ART use has become more efficient and better accepted, and may reflect shifts in the age profiles of patients attending fertility clinics, a faster care-seeking process, and improvements in ART efficiency. Despite this, the likelihood of achieving a spontaneous or ART birth still decreases significantly with age at conception attempt, highlighting persistent biological constraints even in the Norwegian context characterised by advances in infertility treatments. Future research should investigate whether the 2020 policy changes, notably allowing egg donation, may have induced further temporal changes in the occurrence and time to ART births.

## Data Availability

The Trøndelag Health Study (HUNT) has invited persons aged 13-100 years to four surveys between 1984 and 2019. Comprehensive data from more than 140,000 persons having participated at least once and biological material from 78,000 persons are collected. The data are stored in HUNT databank and biological material in HUNT biobank. HUNT Research Centre has permission from the Norwegian Data Inspectorate to store and handle these data. The key identification in the database is the personal identification number given to all Norwegians at birth or immigration, whilst de-identified data are sent to researchers upon approval of a research protocol by the Regional Ethical Committee and HUNT Research Centre. To protect participants’ privacy, HUNT Research Centre aims to limit storage of data outside HUNT databank, and cannot deposit data in open repositories. HUNT databank has precise information on all data exported to different projects and are able to reproduce these on request. There are no restrictions regarding data export given approval of applications to HUNT Research Centre. For more information see: http://www.ntnu.edu/hunt/data.
